# “Impact of aging on maximal oxygen uptake adjusted for lower limb lean mass, total body mass, and absolute values in runners”

**DOI:** 10.1007/s11357-023-00828-z

**Published:** 2023-05-26

**Authors:** Aldo Seffrin, Lavínia Vivan, Vinícius Ribeiro dos Anjos Souza, Ronaldo Alves da Cunha, Claudio Andre Barbosa de Lira, Rodrigo Luiz Vancini, Katja Weiss, Beat Knechtle, Marilia Santos Andrade

**Affiliations:** 1 Postgraduate Program in Translation Medicine, Federal University of São Paulo, São Paulo, Brazil; 2 Human and Exercise Physiology Division, Faculty of Physical Education and Dance, Federal University of Goiás, Goiás, Brazil; 3 Center for Physical Education and Sports, Federal University of Espírito Santo, Espírito Santo, Brazil; 4 Institute of Primary Care, University of Zurich, Zurich, Switzerland; 5 Department of Physiology, Federal University of São Paulo, São Paulo, Brazil

**Keywords:** Aging, V̇O_2_*max*, Performance, Runners, Muscles

## Abstract

Performance in endurance sports decreases with aging, which has been primarily attributed to cardiovascular and musculoskeletal aging; however, there is still no clear information on the factors that are most affected by aging. The aim of this study was to compare two groups of runners (< 50 and > 50 years of age) according to their absolute, weight-adjusted maximal oxygen uptake (V̇O_2_*max*), lower limb lean mass-adjusted V̇O_2_*max*, ventilatory threshold, and respiratory compensation point (RCP). A total of 78 male recreational long-distance runners were divided into Group 1 (38.12 ± 6.87 years) and Group 2 (57.55 ± 6.14 years). Participants were evaluated for body composition, V̇O_2_*max*, VT, and RCP. Group 1 showed higher absolute and body mass-adjusted V̇O_2_*max* (4.60 ± 0.57 l·min^−1^ and 61.95 ± 8.25 ml·kg^−1^·min^−1^, respectively) than Group 2 (3.77 ± 0.56 l·min^−1^ and 51.50 ± 10.22 ml·kg^−1^·min^−1^, respectively), indicating a significant difference (*p* < 0.001, d =  − 1.46 and *p* < 0.001, d =  − 1.16). Correspondingly, Group 1 showed a significantly higher lower limb lean mass–adjusted V̇O_2_*max* (251.72 ± 29.60 ml·kgLM^−1^·min^−1^) than Group 2 (226.36 ± 43.94 ml·kgLM^−1^·min^−1^) (*p* = 0.008, d =  − 0.71). VT (%V̇O_2_*max*) (*p* = 0.19, d = 0.19) and RCP (%V̇O_2_*max*) (*p* = 0.24, d = 0.22) did not differ between the groups. These findings suggest that both variables that are limited by central or peripheral conditions are negatively affected by aging, but the magnitude of the effect is higher in variables limited by central conditions. These results contribute to our understanding of how aging affects master runners.

## Introduction

It is a well-known fact that performance in long-distance running events decreases with increasing age in a curvilinear manner [1]. Peak performance is generally achieved between the ages of 30 and 39 years, and only a modest decline occurs until the age of 50 years [2], coinciding with a significant muscle mass loss (~ 1.4% per year) after the age of 50 years [3].

One of the major determinants of long-distance exercise performance is the maximal oxygen uptake (V̇O_2_*max*), which is defined as the highest rate at which oxygen can be taken up by the lungs, transported by the cardiovascular system, and used by the muscular system [4]. Therefore, V̇O_2_*max* establishes the upper limit of maximal energy production through oxidative pathways [5]. In addition to V̇O_2_*max*, the percentage of V̇O_2_*max* that can be maintained during an endurance run, which is related to the V̇O_2_ measured at lactate threshold, is extremely important for long-distance exercise performance [4]. Finally, the energy demand to run at a given velocity, which has been termed running economy, is also an important determinant of performance, especially among athletes presenting similar V̇O_2_*max* values [6].

V̇O_2_*max*, which is an extremely important measurement reflecting the upper limit of endurance performance, is measured as an absolute rate of oxygen uptake per unit of time (l·min^−1^) in liters per minute, and there is a relative consensus in the literature that the major limiting factor of maximum V̇O_2_*max* is the central condition, i.e., the maximum cardiac output [7]. Although V̇O_2_*max* is measured in liters per minute, it can be expressed as a weight-adjusted rate (ml·kg^−1^·min^−1^), which is more adequate to compare individuals with different body masses [7]. This measure is also influenced by body composition, which becomes worse with increasing fat mass and decreasing lean mass during the aging process [8].

In this context, lower limb lean mass-adjusted V̇O_2_*max*, which has been termed “aerobic muscle quality,” is also essential for endurance performance and is gaining relevance [9]. As it measures the muscular capacity for oxygen consumption [10], lean mass-adjusted V̇O_2_*max* measurements are influenced by several peripheral conditions, such as mitochondrial enzyme levels and capillary density [9].

Although the decrease in aerobic performance with aging coincides with the decrease in V̇O_2_*max* values, the decline in V̇O_2_*max* may be greater than the decline in performance [1]. This situation emphasizes the effects of aging on all the physiological factors that affect aerobic performance, including the percentage of V̇O_2_*max* that can be sustained (i.e., aerobic endurance), which is generally measured using the anaerobic threshold (AT) and the respiratory compensation point (RCP), identified through a cardiopulmonary exercise test (CPET) [11].

Considering that the decline in performance due to aging is not of the same magnitude as the decline in V̇O_2_*max* absolute values, it is interesting to understand the magnitude of the impact of aging on other physiological variables that also affect performance but are limited by peripheral factors (e.g., lean mass-adjusted V̇O_2_*max* and AT) rather than central factors (e.g., absolute V̇O_2_*max* and body mass-adjusted V̇O_2_*max*).

Therefore, the present study was conducted to compare two groups of runners (< 50 and > 50 years of age) according to their absolute, weight-adjusted V̇O_2_*max*, lower limb lean mass-adjusted V̇O_2_*max,* and aerobic endurance variables (ventilatory threshold (VT) and RCP). We hypothesized that the physiological variables that are associated with performance and limited by central factors (absolute and weight-adjusted V̇O_2_*max*) would be more affected by the aging process than the variables that are limited by peripheral factors (aerobic endurance variables and lower limb lean mass-adjusted V̇O_2_*max*). Overall, this study emphasizes the importance of considering both central and peripheral factors in determining the effects of aging on running performance and provides a valuable resource for developing evidence-based training and testing interventions for runners of different ages and experience levels.

## Materials and methods

### Ethical approval

All experimental procedures were reviewed and approved by the Human Research Ethics Committee of the Federal University of Sao Paulo (approval number 4.354.386, October 22, 2020). The participants also signed a consent form after being provided with information regarding the study objectives, experimental procedures, risks, and benefits, as well as the assurance of privacy and confidentiality.

### Participants

In total, 78 recreationally trained male runners were recruited through contact with running consultants, running coaches, training clubs, social media outreach, and partnership with sports medicine units. For analysis purposes, the participants were divided into two age groups, viz., Group 1 (20–49 years, *n* = 49) and Group 2 (50–70 years, *n* = 29). The age of 50 years was selected as the cutoff for both groups because after this age, the V̇O_2_*max* [1] and muscle strength [3] exhibit an accelerated decline. The characteristics of the participants are shown in Table 1.

**Table 1:** Characteristics of the participants

Variables	Group	N	Mean ± SD	*p*-value	Power	Effect size (d)	Effect size CI 95%	
							Lower limit	Upper limit
Age (years)	1	49	38.12 ± 6.87	< 0.001		2.94	2.29	3.59
	2	29	57.55 ± 6.14		1.00			
Body mass (kg)	1	49	74.81 ± 8.66	0.902		0.03	-0.43	0.49
	2	29	75.11 ± 11.09		0.06			
Body height (m)	1	49	1.75 ± 0.07	0.475		-0.17	-0.63	0.29
	2	29	1.74 ± 0.07		0.17			
Body fat (%)	1	49	23.44 ± 4.87	0.001		0.84	0.36	1.32
	2	29	27.60 ± 5.12		0.97			
Total lean mass (kg)	1	49	56.38 ± 5.89	0.029		-0.53	-0.99	-0.06
	2	29	53.27 ± 5.93		0.72			
Lean mass of lower limbs (kg)	1	49	18.36 ± 2.16	0.039		-0.54	-1.01	-0.07
	2	29	17.01 ± 2.98		0.73			
BMI (kg/m^2^)	1	49	24.33 ± 2.10	0.498		0.16	-0.30	0.62
	2	29	24.69 ± 2.29		0.16			
Running experience (years)	1	49	7.41 ± 4.68	< 0.001		1.26	0.76	1.76
	2	29	17.07 ± 11.05		0.99			

Data are presented as mean ± standard deviation (SD). *BMI*, body mass index; *CI*, confidence interval

The participants were required to have participated in a running training program for a minimum of 3 years, at least three times per week, and be aged between 20 and 70 years. To ensure study accuracy, only the participants with a BMI ≤ 29.9 kg/m^2^ were included because obesity can exert a negative impact on the variables under investigation, increase physical exertion, and potentially cause cardiovascular strain. Furthermore, obese individuals may have other health issues that could complicate study outcomes, further justifying their exclusion. In addition, participants should not have any medical contraindications to maximal effort exercise, smoking history, myopathies, systemic inflammatory disease, parenchymal lung disease, cardiomyopathy, peripheral vascular disease, or orthopedic conditions that would limit maximum performance in the assessments. Information regarding the training habits of participants is presented in Table 2. Only the participants who answered “NO” to all PAR-Q questions and did not have any contraindication to participation were eligible for the study. However, those who answered “YES” to at least one question, even if they were asymptomatic, were required to undergo a medical examination. The medical examination evaluated the family history of cardiovascular disease, relevant risk factors for coronary artery disease, risk classification, and signs of cardiovascular and pulmonary disease. Only with the approval of a physician were these individuals included in this study.

**Table 2:** Characteristics of the sample training habits

Variables	Group	N	Mean ± SD	*p*-value	Power	Effect size (d)	Effect size CI 95%	
							Lower limit	Upper limit
Mileage per week (km·week^−1^)	1	48	42.83 ± 25.17	0.292		− 0.23	− 0.69	0.23
	2	28	37.57 ± 17.81		0.25			
Frequency (days·week^−1^)	1	49	4.12 ± 1.52	0.245		− 0.28	− 0.74	0.18
	2	28	3.68 ± 1.63		0.32			
Season’s best 10-km time (min)	1	48	44.79 ± 9.80	0.026		0.66	0.17	1.14
	2	27	53.63 ± 18.36		0.87			

Data are presented as mean ± standard deviation (SD). *CI*, confidence interval

### Study design

This was a cross-sectional study consisting of two visits to the exercise physiology laboratory during the morning period. During the first visit, an anamnesis was conducted using questionnaires, and during the second visit, body composition assessment and CPET were performed. The interval between visits was less than seven consecutive days to avoid any interference of the training routine in the research results. Participants were instructed to avoid engaging in intense exercise in the 24 h preceding their laboratory second visit and avoid eating 2 h before the test, but fasting was not required so that they could follow their regular hydration routine. They should wear suitable clothes and shoes for performing physical activities and avoid using any stimulating substance that could alter their performance in the tests, especially caffeine at least 8 h before the appointments.

### Anamnesis

A custom questionnaire comprising 38 questions was developed to collect personal information, anthropometric data, and training characteristics. Participant’s body height and body mass were measured using a calibrated wall stadiometer and a balance (Filizola® São Paulo, Brazil) to the nearest 0.1 cm and 0.1 kg, respectively. The questionnaire consisted of the following three open questions: (1) How many years have you been running? (2) How many hours per week do you train? (3) Have you ever experienced any medical condition that could prevent you from engaging in physical activity in the last year? The participants also completed the Physical Activity Readiness Questionnaire (PAR-Q) [12].

### Assessment of body composition

Body composition was evaluated using the dual-emission X-ray absorptiometry system (Software version 12.3, Lunar DPX, Wisconsin, USA). This technique involves minimal radiation exposure and is reliable for evaluating body composition [13]. Body mass, body fat, and lean body mass were measured for the whole body as well as for the lower and upper limbs and trunk. Lean mass and fat mass were expressed in absolute (kg) and relative (%) values.

### Cardiopulmonary exercise testing

The CPET was conducted on a treadmill (Imbrasport, ATL, Brazil), and the collected data were used to determine VT, RCP, and metabolic responses. During the test, the participant’s physiological responses to exercise were measured using a metabolic system (Quark PFT; Cosmed®, Rome, Italy), which is a valid, accurate, and reliable tool for evaluating the abovementioned outcomes [14]. This system comprises a respiratory airflow sensor and rapid-response O_2_ and CO_2_ gas analyzers that were heat-adjusted and compensated for variations in barometric pressure, temperature, and relative humidity.

Breath samples were collected using a silicone face mask (V2Mask, Hans Rudolph Inc., USA), fixed with a cap supplied by the manufacturer, connected to a breathing valve, and transported to the analyzers through a semi-permeable Nafion® (synthetic polymer consisting of sulfhydryl residues) sample line that allowed rapid equilibrium with water vapor in the environment. Data processing was performed using the designated software (PFT, Cosmed®, Rome, Italy), compatible with the Windows® platform (USA). Calibration was performed before each test according to the manufacturer’s guidelines.

All evaluations started with 1 min of rest. If there was evidence of hyperventilation, which indicated anxiety and/or discomfort with the laboratory environment, the rest period was adjusted. This was followed by 3 min of warm-up at a constant speed and 3 min of exercise at 1.7 mph at a 10% grade. After the warm-up period, the test grade and speed were increased according to the Ellestad protocol [15]. The collection of gases for analysis was performed for each breath and then normalized every 20 s.

During the exercise period, all participants were encouraged to perform maximal progressive exercise until volitional exhaustion, as evaluated by symptoms such as dyspnea, severe fatigue, muscle pain, and inability to continue the test. The recovery period was 2 min, during which metabolic, cardiovascular, and respiratory variables were continuously monitored.

VT and RCP were determined using the ventilatory equivalents method [16]. VT was defined as the oxygen consumption corresponding to an effort intensity above in which a first increase in V̇E/V̇O_2_ is observed, i.e., a nonlinear increase in V̇E/V̇O_2_ with a concomitant increase in positive end-expiratory pressure of O_2_ (PEEPO_2_) and without an increase in V̇E/V̇CO_2_. RCP was defined as the oxygen consumption corresponding to an effort intensity above which an increase in V̇E/V̇CO_2_ with a concomitant decrease in positive end-expiratory pressure of CO_2_ (PEEPCO_2_) is observed.

V̇O_2_*max* was determined as the oxygen consumption achieved during maximal exercise intensity that could not be increased by < 150 ml·min^−1^ despite further increases in exercise intensity [17, 18]. If the participant did not meet this requirement, ventilatory, metabolic, and perceived exertion ratings were used to confirm that the test was maximal. These ratings consisted of the respiratory exchange ratio (RER) ≥ 1.10, (i.e., the ratio of the amount of CO_2_ being produced to the amount of O_2_ being consumed (V̇CO_2_/V̇O_2_)), if the participant reached the age-predicted maximum heart rate, and Borg’s perceived exertion scale = 20 [19]. Two experienced investigators independently identified VT and RCP. When there was no agreement on the independent identification, a third investigator settled the analysis. V̇O_2_*max* was measured as the absolute value (l·min^−1^), and then the total body mass-adjusted V̇O_2_*max* (ml·kg^−1^·min^−1^) and lower limb lean mass-adjusted V̇O_2_*max* (ml·kgLM^−1^·min^−1^) were calculated.

### Statistical analysis

Descriptive data were expressed as mean, standard deviation, effect sizes, and effect size confidence intervals. Sample size was calculated considering a significant effect size of ≥ 0.51, a significance level of 0.05, and a power of 0.8. The calculation revealed that 98 subjects (49 per group) were required, but due to the COVID-19 outbreak and social distancing requirements, the planned data collection for the older age group could not be completed. An a posteriori analysis was performed based on the achieved effect size for the primary outcome (lower limb lean mass-adjusted V̇O_2_*max* expressed as ml·kgLM^−1^·min^−1^), which was 0.71, maintaining a significance level of 0.05 and a power of 0.8. The analysis revealed that at least 26 subjects per group would be sufficient. All data followed normal distribution according to the Shapiro–Wilk test, and homogeneity of variances was checked using Levene’s test. Differences between the two groups were investigated using Welch’s *t*-test because it performs better than Student’s *t*-test whenever sample sizes are different [20]. The measures of effect size for differences between groups were determined by calculating the mean difference between the two groups and then dividing the result by the pooled standard deviation. The magnitude of any change was judged based on the following criteria: d < 0.2 was considered to have no effect, 0.2 ≤ d < 0.5 was considered a small effect size, 0.5 ≤ d < 0.8 represented a medium effect size, 0.8 ≤ d < 1.3 indicated a large effect size, and d ≥ 1.3 was a very large effect size [21]. The level of significance was set at 0.05. All statistical analyses were performed using SPSS version 26.0 (SPSS, Inc., Chicago, IL, USA).

## Results

Regarding running experience, as intentionally selected, older age runners (Group 2) had significantly more years of running experience (*p* < 0.001; d = 1.26 (large)). Moreover, the groups differed in terms of body composition, with significant differences found in the percentage of body fat (*p* = 0.001, d = 0.84 (large)), total lean mass, and lean mass of the lower limbs (*p* = 0.029, d =  − 0.53 (medium), and *p* = 0.039, d =  − 0.54 (medium), respectively). However, no differences were found in terms of total body mass (*p* = 0.902, d = 0.03), body height (*p* = 0.475, d =  − 0.17), or BMI (*p* = 0.498, d = 0.16); the data are presented in Table 1.

To compare the training habits of participants, we compared their total mileage per week and number of running sessions per weekday and found no significant differences between the two groups (*p* = 0.292, d =  − 0.23 (small), and *p* = 0.245, d =  − 0.28 (small), respectively). However, the best 10 km time of the season reported by the participants showed a significant difference favoring the younger age group (*p* = 0.026, d = 0.66 (medium)); these data are shown in Table 2.

A comparison of the cardiorespiratory parameters obtained in the CPET between the groups is presented in Table 3.

**Table 3:** Differences between cardiorespiratory variables obtained in CEPT

Variables	Group	N	Mean ± SD	*p* -value	Power	Effect size (d)	Effect size CI 95%	
							Lower limit	Upper limit
V̇O_2_*max* (l·min^−1^)	1	49	4.60 ± 0.57	< 0.001	0.99	− 1.46	− 1.97	− 0.94
	2	29	3.77 ± 0.56					
V̇O_2_*max* (ml·kg^−1^·min^−1^)	1	49	61.95 ± 8.25	< 0.001	0.99	− 1.16	− 1.65	− 0.66
	2	29	51.50 ± 10.22					
V̇O_2_*max* (ml·kgLM^−1^·min^−1^)	1	49	251.72 ± 29.60	0.008	0.91	− 0.71	− 1.19	− 0.24
	2	29	226.36 ± 43.94					
V̇O_2_ VT (ml·kg^−1^·min^−1^)	1	49	43.44 ± 7.82	0.001	0.96	− 0.82	− 1.30	− 0.34
	2	29	37.06 ± 7.73					
VT (%V̇O_2_*max*)	1	49	70.18 ± 8.60	0.271	0.26	0.24	− 0.22	0.70
	2	29	72.07 ± 6.36					
HR VT (%HR*max*)	1	49	84.45 ± 5.43	0.385	0.22	− 0.21	− 0.67	0.25
	2	29	83.33 ± 5.51					
V̇O_2_ RCP (ml·kg^−1^·min^−1^)	1	49	54.76 ± 7.16	< 0.001	0.99	− 1.12	− 1.61	− 0.63
	2	29	46.01 ± 8.84					
RCP (%V̇O_2_*max*)	1	49	88.59 ± 5.47	0.403	0.20	0.19	− 0.27	0.66
	2	29	89.64 ± 5.24					
HR RCP (%HR*max*)	1	49	94.10 ± 2.63	0.126	0.50	− 0.39	− 0.85	0.07
	2	29	92.96 ± 3.34					

Data are presented as mean ± standard deviation (SD). V̇*O*_*2*_, oxygen uptake; *max*, maximum; *HR*, heath rate; *VT*, ventilatory threshold; *RCP*, respiratory compensation point; *LM*, lean mass

## Discussion

This study explored the differences in absolute, total body mass-adjusted, and lower limb lean mass-adjusted V̇O_2_*max*, VT, and RCP between two age groups of runners, i.e., < 50 and > 50 years. Our hypothesis was that the variables limited by central conditions, such as absolute and relative to body mass V̇O_2_*max* values, would be more affected by aging than the variables limited by peripheral conditions, such as relative to lean mass V̇O_2_*max*, VT, and RCP.

The major findings were as follows: (i) older age runners showed lower absolute and total body mass-adjusted V̇O_2_*max* (effect sizes were very large and large, respectively) than younger age runners; (ii) older age runners showed lower limb lean mass-adjusted V̇O_2_*max* (effect size was medium) than younger age runners; and (iii) VT (in %V̇O_2_*max*) and RCP (in %V̇O_2_*max*) showed no differences between older and younger age runners. Therefore, our initial hypothesis has been confirmed.

V̇O_2_*max* reflects an individual’s aerobic exercise capacity, which is a key indicator of physical fitness [22]. According to the Fick equation [23, 24], V̇O_2_*max* = maximal cardiac output (CO) × maximal oxygen arteriovenous difference (dif(a-v)O_2_), implying that a higher ability of the respiratory system to extract oxygen from the air, a higher ability of the cardiac system to transport oxygen to the exercising muscles, and a higher ability of the skeletal muscles to utilize oxygen, resulting in higher V̇O_2_*max* [7]. Although all these systems affect V̇O_2_*max*, the cardiovascular system, specifically cardiac systolic and diastolic function, is considered the central hub [25].

Our findings showed significantly lower V̇O_2_*max* (l·min^−1^) in runners aged > 50 years than in runners aged < 50 years, with a very large effect size. This decline in absolute V̇O_2_*max* in aging individuals is anticipated because there is an undeniable reduction in maximal CO with age [26, 27], causing a reduction in V̇O_2_*max*.

Aging also exerts a negative effect on muscle mass. A review by Mitchel et al. (2012) [28] showed that the median decline in muscle mass throughout an individual’s lifespan is 0.47% per year in men. Therefore, older age individuals have lower metabolically active tissue (skeletal muscle mass), which negatively impacts V̇O_2_*max* [29]. Although the older age runner group showed lower V̇O_2_*max* values than the younger age runner group, their V̇O_2_*max* (3.77 ± 0.56 l·min^−1^) was higher than the literature reference value for nonathletes aged 30 years (3.2 l·min^−1^) [30].

Despite the importance of absolute V̇O_2_*max*, this variable does not consider the body characteristics of an individual. Hence, the total body mass-adjusted V̇O_2_*max* shows a higher association with endurance performance [22], general health, and functional capacity [29]. In this context, elite male athletes typically have a V̇O_2_*max* between 70 and 85 ml·kg^−1^·min^−1^ [31], and subjects with < 14 ml·kg^−1^·min^−1^ are indicated for heart transplantation [32]. The cutoff for an independent lifestyle has been considered 18 and 15 ml·kg^−1^·min^−1^ in men and women, respectively [33].

The results of the present study also demonstrated a significant difference between the two age groups according to V̇O_2_*max* relative to body mass. Although the effect size was classified as large, it was lower than the effect size for absolute V̇O_2_*max* values. Despite the evident reduction in total body mass-adjusted V̇O_2_*max*, the older age runner group showed values much higher than expected for their age group, considering the literature data for healthy adults. According to a review based on 62 previous studies [30], V̇O_2_*max* values > 46 ml·kg^−1^·min^−1^ are considered excellent for men aged 50–55 years, and the older age group in the present study showed a mean V̇O_2_m*ax* value of 51.5 ml·kg^−1^·min^−1^. These data indicate that despite the decrease in V̇O_2_*max* with age, even in runners, the runners may live longer with higher V̇O_2_*max* values, which implies more years of independent life.

Another insightful method for normalization is expressing V̇O_2_*max* adjusted by the lean mass. Lean mass (skeletal muscle mass) is the largest component of the fat-free body mass in humans [34], which is required for athletic performance and also for an independent lifestyle for elderly individuals [28]. This variable should also be evaluated to examine physical fitness because reduced muscular aerobic capacity also contributes to reduced whole-body V̇O_2_*max* absolute values [35]. As lean mass-adjusted V̇O_2_*max* reflects the amount of oxygen consumed per kilogram of skeletal muscle, it has been termed “aerobic muscle quality” [9]. There are several suggested limiting factors for this variable, which are primarily peripheral conditions, such as intramuscular adipose tissue, capillary density, structural composition, and mitochondrial density [9, 36]. The older age group in this study showed significantly lower limb lean mass-adjusted V̇O_2_*max* than the younger age group; however, the effect size was medium, which was lower than the effect size for absolute and body mass-adjusted V̇O_2_*max*, evidencing a smaller impact of aging on the peripheral condition (muscular aerobic capacity) than on the central condition (CO).

Similarly, both VT and RCP (%V̇O_2_*max*) showed no significant differences between the two age groups. Similar to lower limb lean mass-adjusted V̇O_2_*max*, the percentage of V̇O_2_*max* that can be sustained is primarily associated with muscular conditions (such as the percentage of type I muscle fibers, mitochondrial level, motor unit recruitment, and firing rates) [7]. This supports the finding that the effects of aging are more pronounced on variables constrained by the central condition than on those constrained by the peripheral condition among runners.

Data from the literature show that the decline in V̇O_2_*max* due to aging is greater than the decrease in performance [1]. The results of the present study partially explain this phenomenon, because other performance-related variables, such as peripheral oxidative capacity, are less affected by aging than absolute V̇O_2_*max.*

## Study limitations

There are some limitations in this study that must be considered. This study used a cross-sectional design, implying that the findings merely provide a momentary depiction of the participants’ physiological status. Further research implementing longitudinal studies is necessary to establish how these variables evolve over time. Moreover, the use of self-reported questionnaires for anamnesis is a potential source of recall bias.

Most importantly, another potential limitation of this study is the suggestion and use of comparisons of rates or proportions between the two groups to draw conclusions about differences in performance or outcomes. This approach can be influenced by rate difference bias, which occurs when a small difference in the underlying rates of the two groups is amplified using rates or proportions in the statistical analysis. This can cause an overestimation or underestimation of the true difference in performance or outcomes between the groups.

## Practical applications

This study provides valuable insights into the differences in V̇O_2_*max* among young and old age runners with varying levels of experience. The results indicate that although absolute V̇O_2_*max* declines with age, V̇O_2_*max* relative to the lean mass of lower limbs is less affected in older age runners compared with absolute values. Our study has practical applications in designing training programs for runners of different ages and experience levels. It also adds to our knowledge of the effect of aging on the physiological variables that are strongly associated with health status. Coaches, trainers, and researchers could use these data to develop exercise programs that emphasize central or peripheral adaptations in runners, depending on the major physiological system affected by the aging process.

## Conclusions

This study suggests that although the variables that affect endurance performance, which are limited by the central condition (absolute V̇O_2_*max*), are severely negatively affected by aging, other variables that are limited by the peripheral condition (lower limb lean mass-adjusted V̇O_2_*max*) are less affected by aging. Despite the limitation of the cross-sectional design used in this study, the findings could provide useful insights for understanding the effects of aging on the physiological determinants of aerobic performance and for the development of effective training programs and interventions for runners, particularly for master athletes who constitute an increasing category of practitioners in recent decades.

## Data Availability

We will make available the data upon request.
